# Supplementary material for: Hysteresis of tropical forests in the 21st century
Source: Nat Commun. 2020 Oct 5;11:4978. doi: 10.1038/s41467-020-18728-7 (PMC7536390; doi:10.1038/s41467-020-18728-7)
Supplement: Supplementary file 1 — Supplementary Information [file 41467_2020_18728_MOESM1_ESM.pdf]

Supplementary Information

Hysteresis of tropical forests in the 21st century

Staal et al.

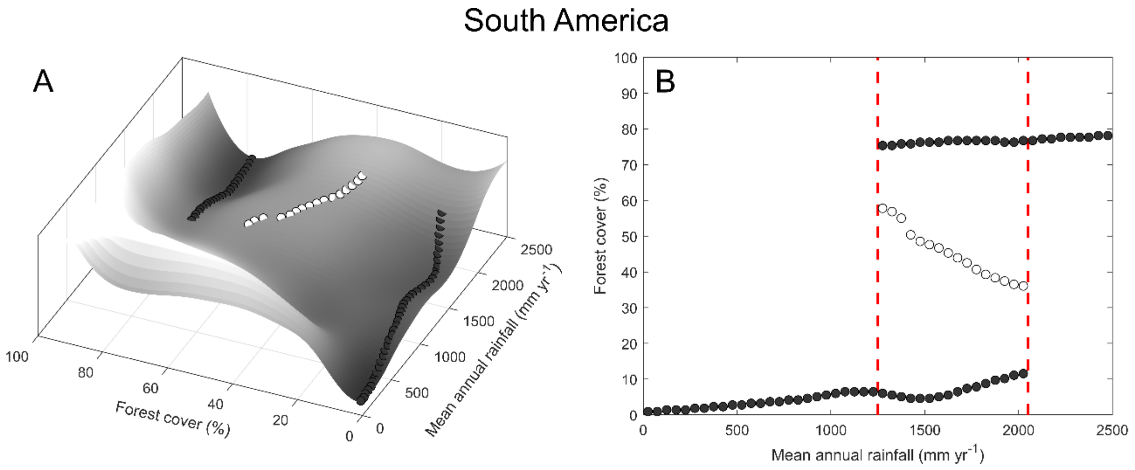

Supplementary Figure 1: Empirically derived bifurcation pattern for forest cover against mean annual rainfall in South America. A) Three-dimensional stability landscape, in which the depth qualitatively represents the potential energy of the state as inferred from forest cover data on 30 m resolution across the continent. Solid dots correspond to significant valleys and represent stable states, and open dots correspond to significant peaks and represent unstable states. B) Two-dimensional bifurcation plot, in which the red lines demarcate the bistability range between 1250−2050 mm yr^{-1} at which forest and nonforest, a savanna-like state of low forest cover, are considered alternative stable states.

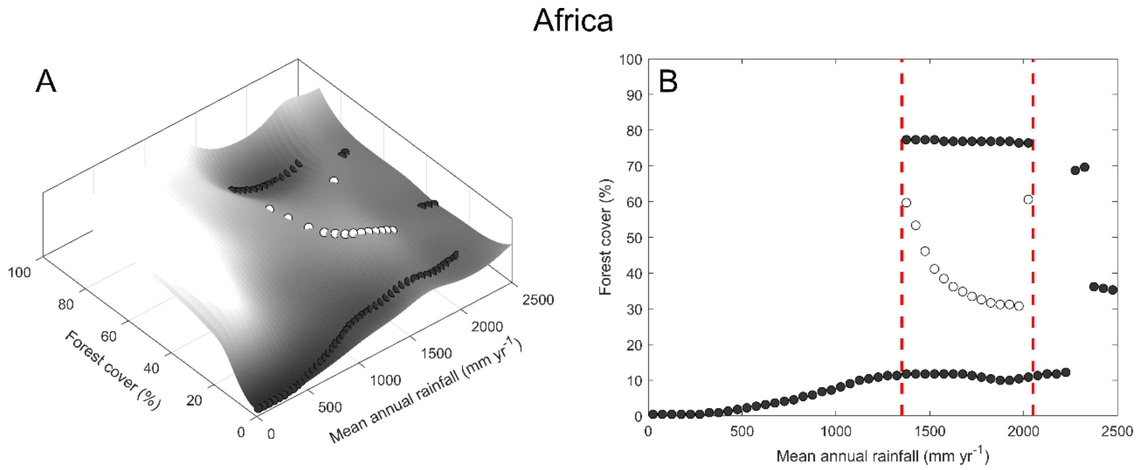

Supplementary Figure 2: Empirically derived bifurcation pattern for forest cover against mean annual rainfall in Africa. A) Three-dimensional stability landscape, in which the depth qualitatively represents the potential energy of the state as inferred from forest cover data on 30 m resolution across the continent. Solid dots correspond to significant valleys and represent stable states, and open dots correspond to significant peaks and represent unstable states. B) Two-dimensional bifurcation plot, in which the red lines demarcate the bistability range between 1350–2050 mm yr^{-1} at which forest and nonforest, a savanna-like state of low forest cover, are considered alternative stable states. Above ~2000 mm yr^{-1} rainfall, data points are scarce (see Supplementary Fig. 7) and dominated by deforestation. Bistability is assumed until the point where the analysis was possible with a sufficient amount of data points (2050 mm yr^{-1}).

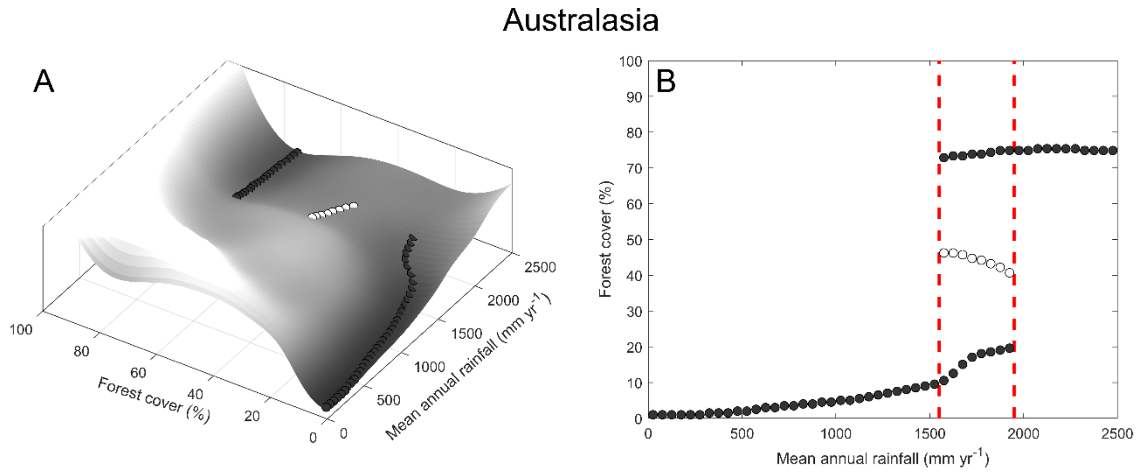

Supplementary Figure 3: Empirically derived bifurcation pattern for forest cover against mean annual rainfall in Australasia. A) Three-dimensional stability landscape, in which the depth qualitatively represents the potential energy of the state as inferred from forest cover data on 30 m resolution across the continent. Solid dots correspond to significant valleys and represent stable states, and open dots correspond to significant peaks and represent unstable states. B) Two-dimensional bifurcation plot, in which the red lines demarcate the bistability range between 1550−1950 mm yr^{-1} at which forest and nonforest, a savanna-like state of low forest cover, are considered alternative stable stats.

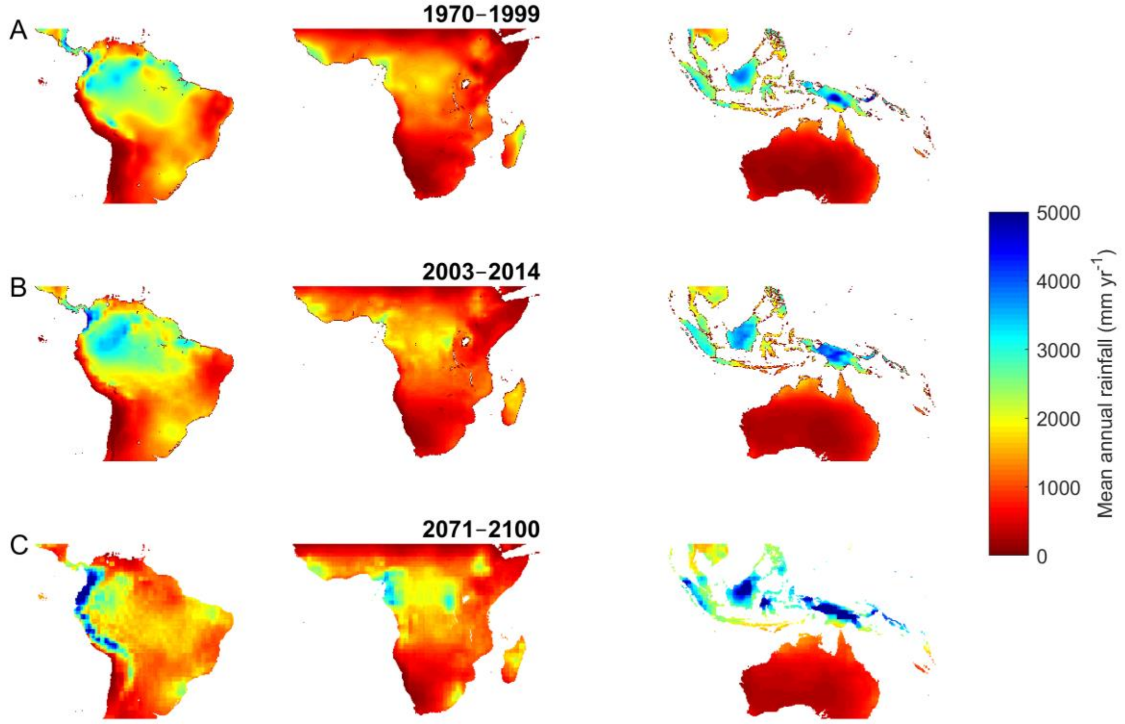

Supplementary Figure 4: Mean annual rainfall (mm yr^{-1}) across the tropics during 1970–1999, 2003–2014 and 2071–2100. A) 1970–1999, the climatic period used to determine the bifurcation diagrams for forest cover (Supplementary Figs. 1–3). B) 2003–2014, the period for which the hydrological simulations were performed. C) 2071–2100 according to the average of seven CMIP6 model runs for the SSP5-8.5 scenario.

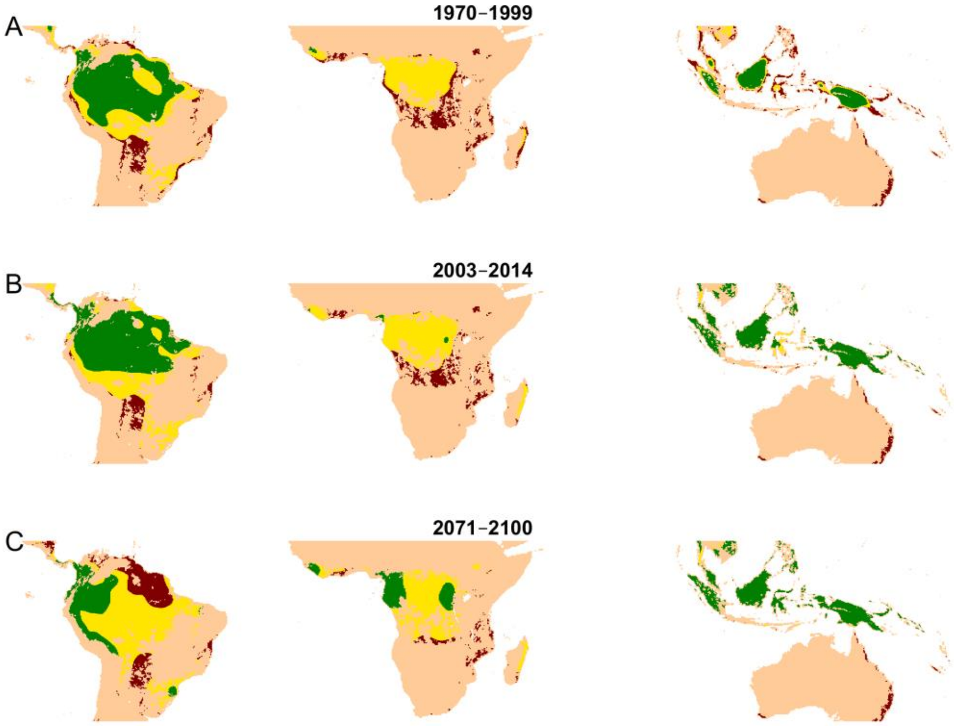

Supplementary Figure 5: Forests (forest cover ≥ 50%) across the tropics classified based on the bifurcation diagrams against mean annual rainfall (Fig. 1; Supplementary Figs. 1−3). A) 1970–1999, the climatic period used to determine the bifurcation diagrams for forest cover. At these rainfall levels and given the forest distribution of 1999, in South America, 4.88 million km^2 of forest is stable; in Africa, 250.000 km^2 is stable; and in Australasia, 2.13 million km^2 is stable. B) 2003–2014, the period for which the hydrological simulations were performed. At these rainfall levels and given the forest distribution of 1999, in South America, 4.93 million km^2 of forest is stable; in Africa, 150.000 km^2 is stable; and in Australasia, 2.12 million km^2 is stable. C) 2071–2100 according to the average of seven CMIP6 model runs for the SSP5-8.5 scenario. At these rainfall levels and given the forest distribution of 1999, in South America, 1.79 million km^2 of forest is stable; in Africa, 1.07 million km^2 is stable; and in Australasia, 2.21 million km^2 is stable.

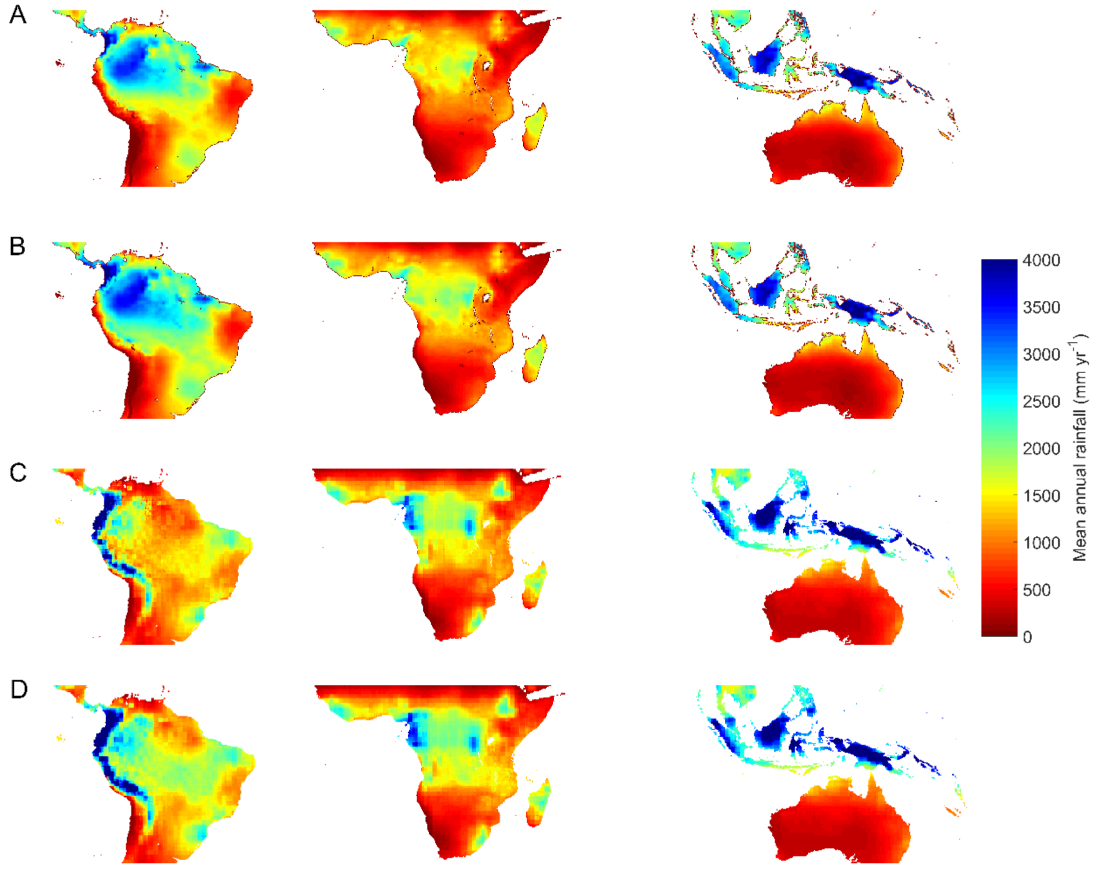

Supplementary Figure 6: Mean annual rainfall levels depending on the hysteresis of tropical forests under recent climate (2003–2014) and under the SSP5-8.5 scenario for 2071–2100. A) Mean annual rainfall (mm yr^{-1}) at minimal forest extent; B) Mean annual rainfall (mm yr^{-1}) at maximal forest extent; C) Mean annual rainfall (mm yr^{-1}) at minimal forest extent for the late 21st century; D) Mean annual rainfall (mm yr^{-1}) at maximal forest extent for the late 21st century.

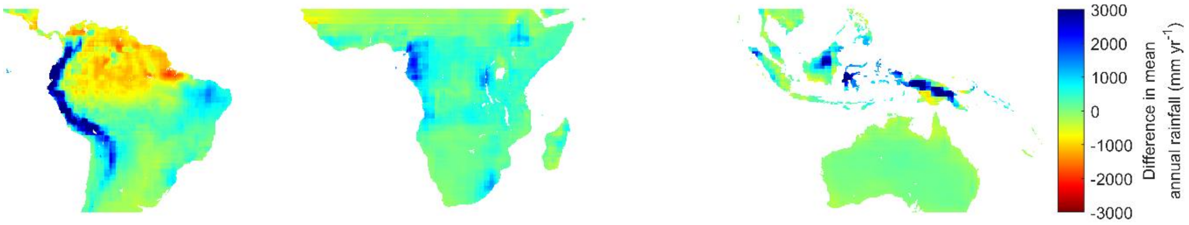

Supplementary Figure 7: Difference in mean annual rainfall levels in mm yr^{-1} between the recent climate (2003–2014) at minimal forest and the SSP5-8.5 scenario for 2071–2100 at maximal forest extent. The color scale was cut off at the high end at 3000 mm yr^{-1}.

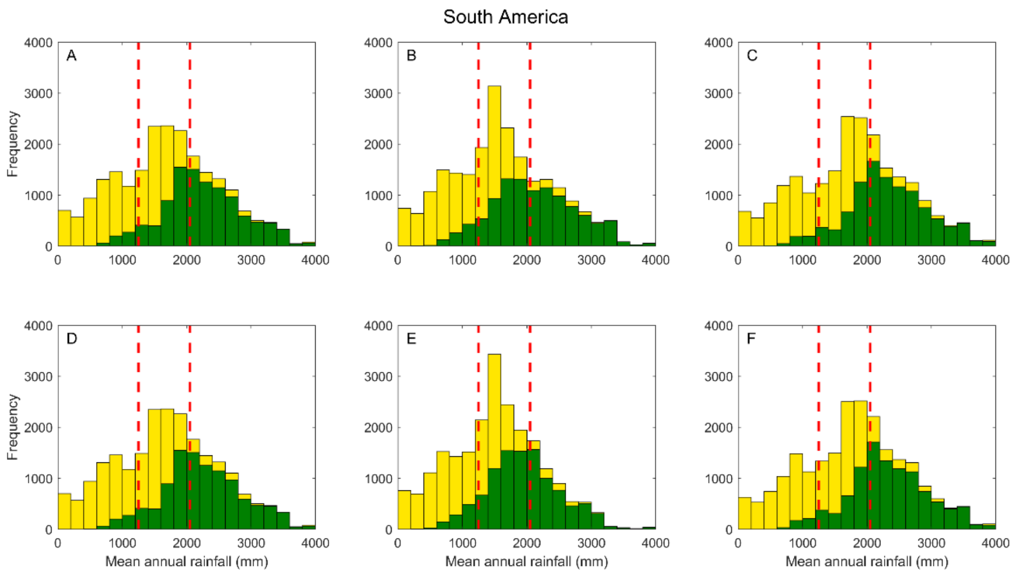

Supplementary Figure 8: Histograms of mean annual rainfall values on 0.25° resolution in South America for forest (green; forest cover ≥ 50%) and nonforest (yellow; forest cover < 50%). A) 2003−2014; B) 2003−2014 in case of a minimal area of stable forest; C) 2003−2014 in case of a maximal area of stable forest; D) 2071−2100 under the SSP5-8.5 scenario, averaged over CMIP6 models; E) 2071−2100 under the SSP5-8.5 scenario, averaged over CMIP6 models, in case of a minimal area of stable forest; F) 2071−2100 under the SSP5-8.5 scenario, averaged over CMIP6 models, in case of a maximal area of stable forest. The red lines indicate the bistability range (see Methods, Supplementary Fig. 1).

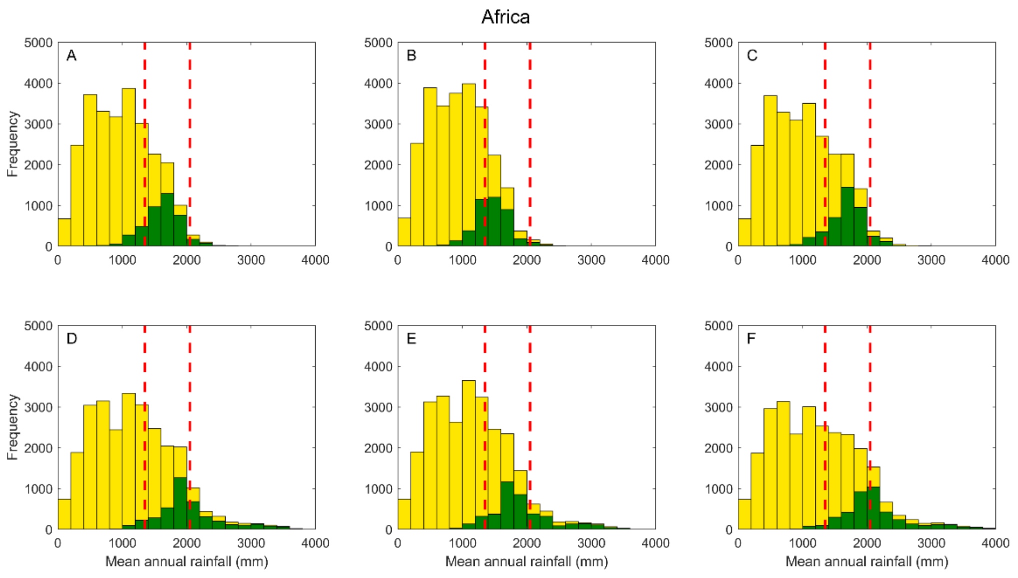

Supplementary Figure 9: Histograms of mean annual rainfall values on 0.25° resolution in Africa for forest (green; forest cover ≥ 50%) and nonforest (yellow; forest cover < 50%). A) 2003−2014; B) 2003−2014 in case of a minimal area of stable forest; C) 2003−2014 in case of a maximal area of stable forest; D) 2071−2100 under the SSP5-8.5 scenario, averaged over CMIP6 models; E) 2071−2100 under the SSP5-8.5 scenario, averaged over CMIP6 models, in case of a minimal area of stable forest; F) 2071−2100 under the SSP5-8.5 scenario, averaged over CMIP6 models, in case of a maximal area of stable forest. The red lines indicate the bistability range (see Methods, Supplementary Fig. 2).

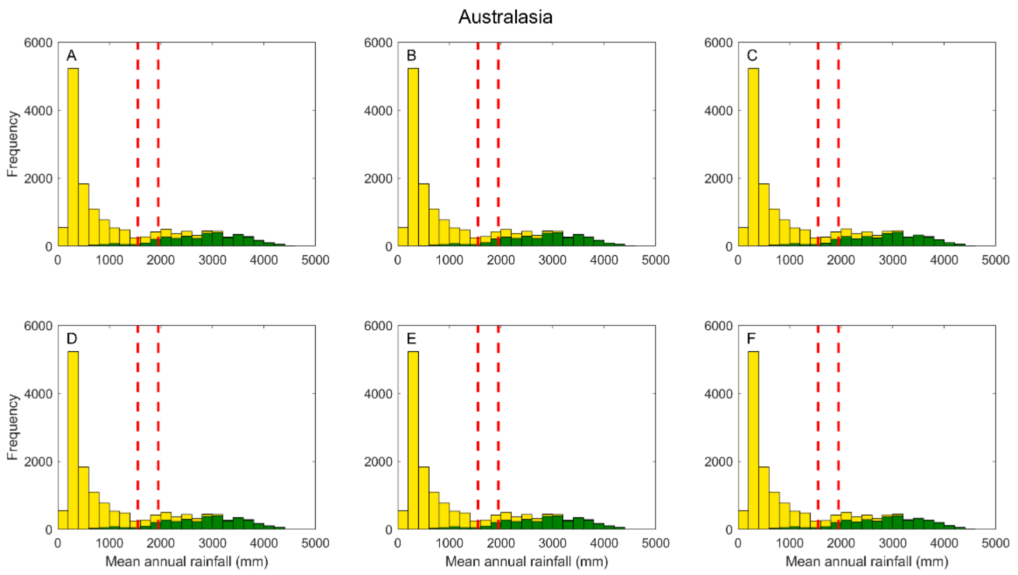

Supplementary Figure 10: Histograms of mean annual rainfall values on 0.25° resolution in Australasia for forest (green; forest cover ≥ 50%) and nonforest (yellow; forest cover < 50%). A) 2003−2014; B) 2003−2014 in case of a minimal area of stable forest; C) 2003−2014 in case of a maximal area of stable forest; D) 2071−2100 under the SSP5-8.5 scenario, averaged over CMIP6 models; E) 2071−2100 under the SSP5-8.5 scenario, averaged over CMIP6 models, in case of a minimal area of stable forest; F) 2071−2100 under the SSP5-8.5 scenario, averaged over CMIP6 models, in case of a maximal area of stable forest. The red lines indicate the bistability range (see Methods, Supplementary Fig. 3).

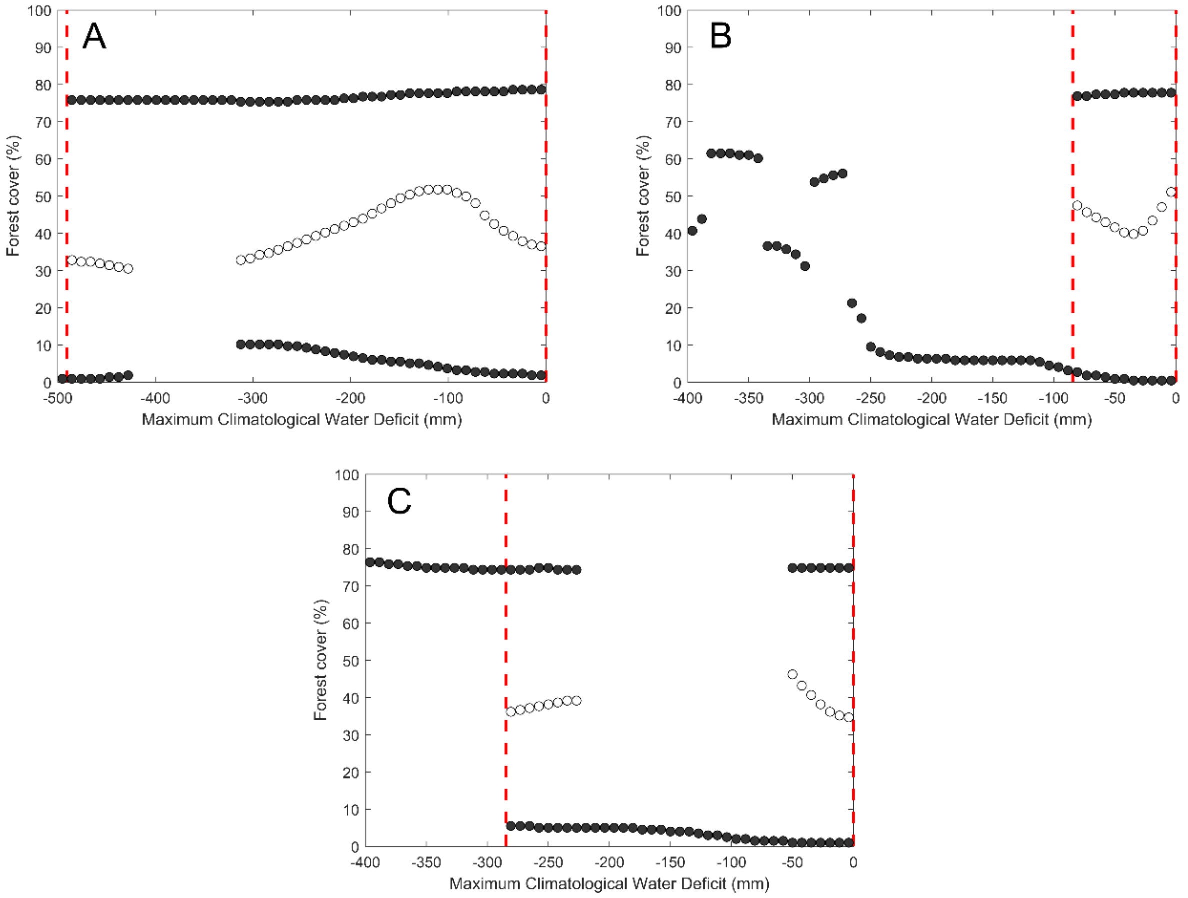

Supplementary Figure 11: Empirical bifurcation plots for forest cover against Maximum Climatological Water Deficit (MCWD in mm). Solid dots represent stable states, and open dots represent unstable states, as inferred from forest cover data on 30 m resolution across each continent (also see Supplementary Figs. 1−3). A) South America, with the red lines demarcating the bistability range between -490−0 mm at which forest and nonforest, a savanna-like state of low forest cover, would be alternative stable states. B) Africa, with the red line demarcating. the bistability range between -80−0 mm at which forest and nonforest, a savanna-like state of low forest cover, would be alternative stable states. C) Australasia, with the red line demarcating the bistability range between -290−0 mm at which forest and nonforest, a savanna-like state of low forest cover, would be alternative stable states.

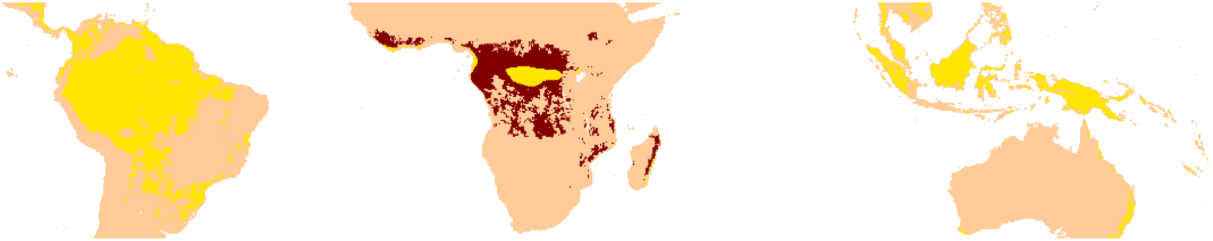

Supplementary Figure 12: Forests (forest cover ≥ 50%) across the tropics in the early 21st century classified based on the bifurcation diagrams against Maximum Climatological Water Deficit (Supplementary Fig. 11). The colors accord to those in Fig. 1 and Supplementary Fig. 5.

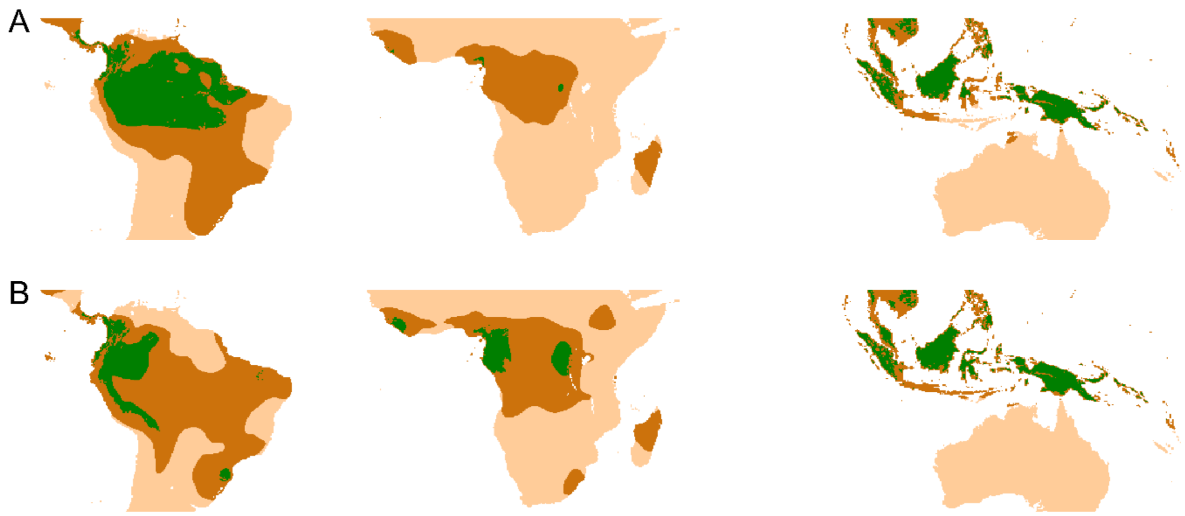

Supplementary Figure 13: Hysteresis of forest cover in the tropics under early 21st century climate and under late 21st century climate, without adjusting rainfall levels for forest-induced moisture recycling. A) Minimal (green) and maximal (brown) forest distributions under early 21st century climate. B) Minimal (green) and maximal (brown) forest distributions under late 21st century climate.

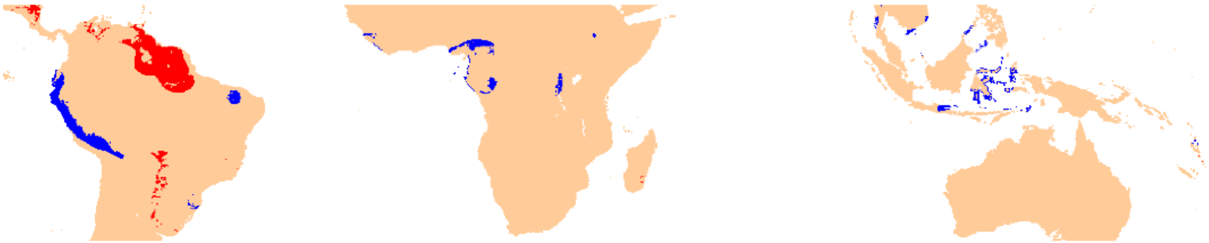

Supplementary Figure 14: Tipping points between forests and savannas in the 21st century based on changes in mean annual rainfall as projected in the SSP5-8.5 scenario from CMIP6 models. Red: from forest to nonforest; blue: from nonforest to forest.

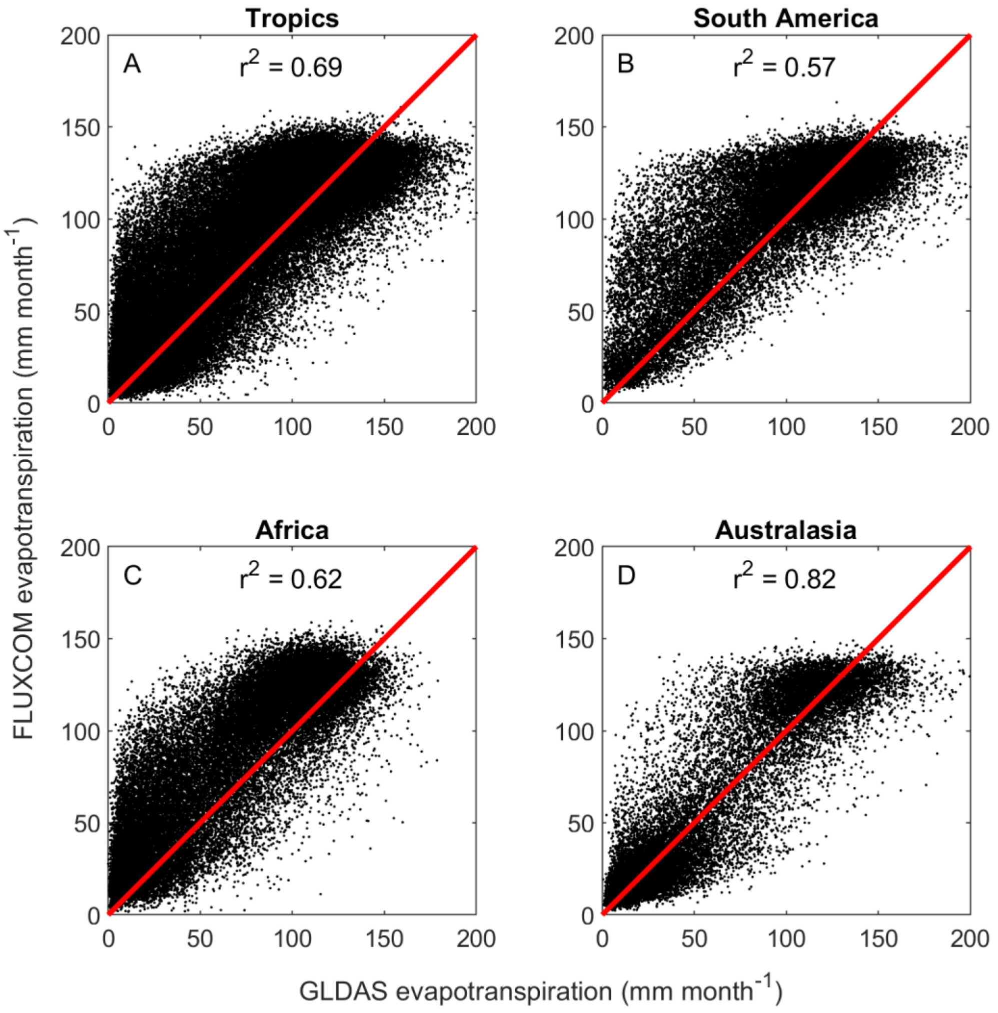

Supplementary Figure 15: Monthly evapotranspiration in mm month^{-1} for 2003–2014 in the GLDAS dataset (used in this study) and the FLUXCOM dataset at 0.25^{o} resolution. The r^2 refer to the concordance correlation, i.e. the correspondence along the 1:1 line. A) Tropics, where r^2 = 0.69. B) South America, where r^2 = 0.57. C) Africa, where r^2 = 0.62. D) Australasia, where r^2 = 0.82.

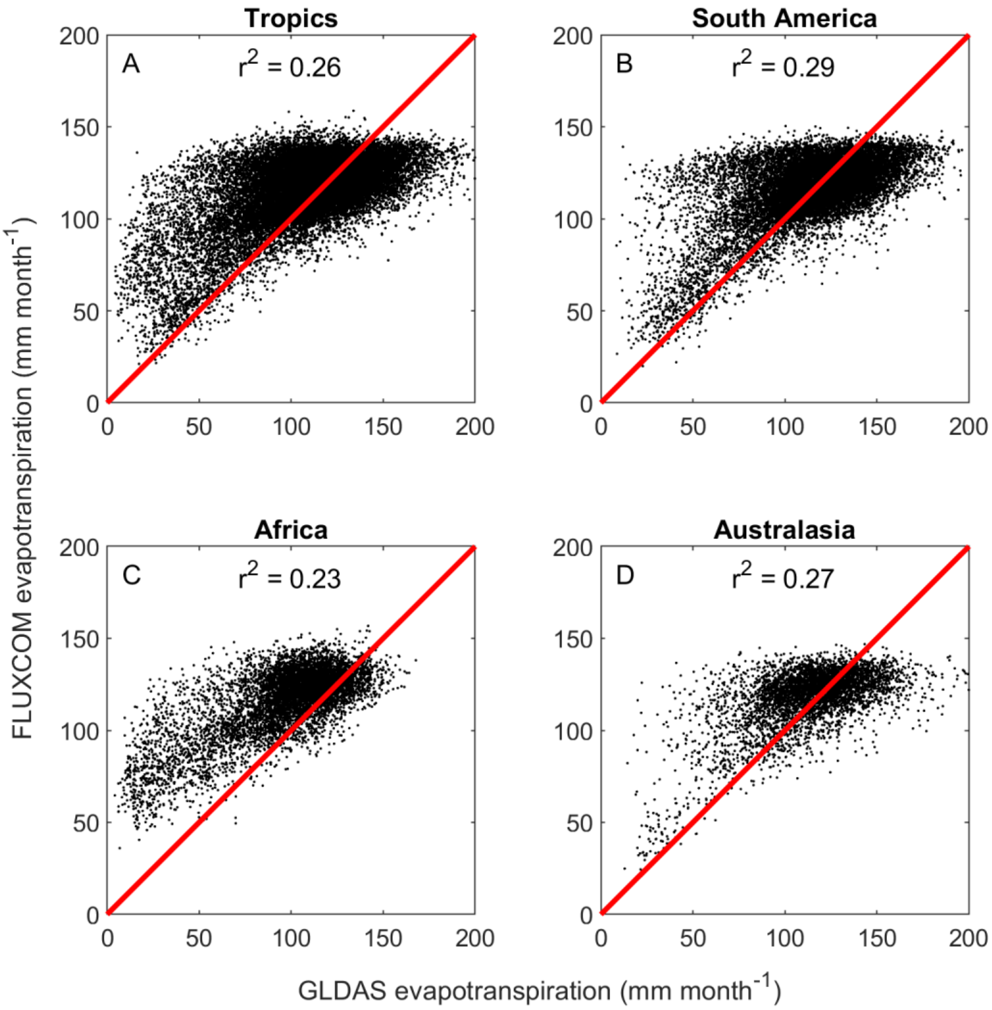

Supplementary Figure 16: Monthly evapotranspiration in mm month^{-1} for 2003–2014 in the GLDAS dataset (used in this study) and the FLUXCOM dataset at 0.25° resolution, for only the forested cells (forest cover ≥ 50%). The r^2 refer to the concordance correlation, i.e. the correspondence along the 1:1 line. A) Tropics, where r^2 = 0.26. B) South America, where r^2 = 0.29. C) Africa, where r^2 = 0.23. D) Australasia, where r^2 = 0.27.

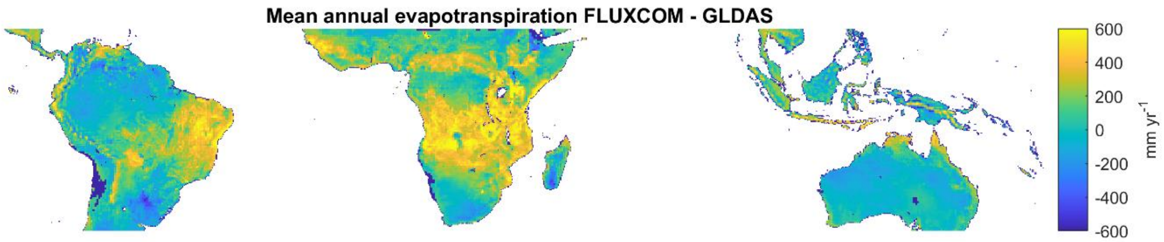

Supplementary Figure 17: Difference in mean annual evapotranspiration (mm yr^{-1}) between GLDAS (used in this study). Positive values indicate that FLUXCOM estimates larger evapotranspiration values, whereas negative values indicate that GLDAS estimates larger evapotranspiration.

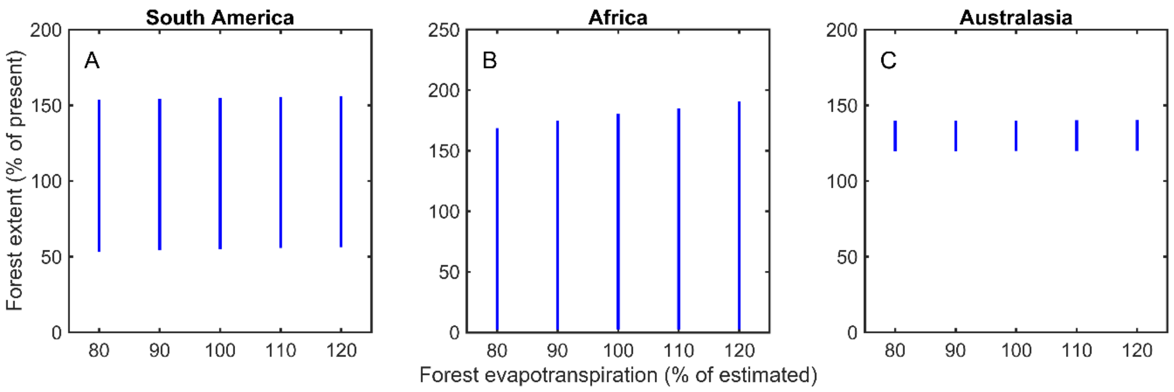

Supplementary Figure 18: Sensitivity of forest hysteresis (% of present extent) to forest evapotranspiration (% of estimated). The range of the blue bars depicts the hysteresis for 80%, 90%, 100%, 110%, and 120% of estimated forest evapotranspiration. Results are based on simulations for 2003 only. A) South America. B) Africa. C) Australasia.

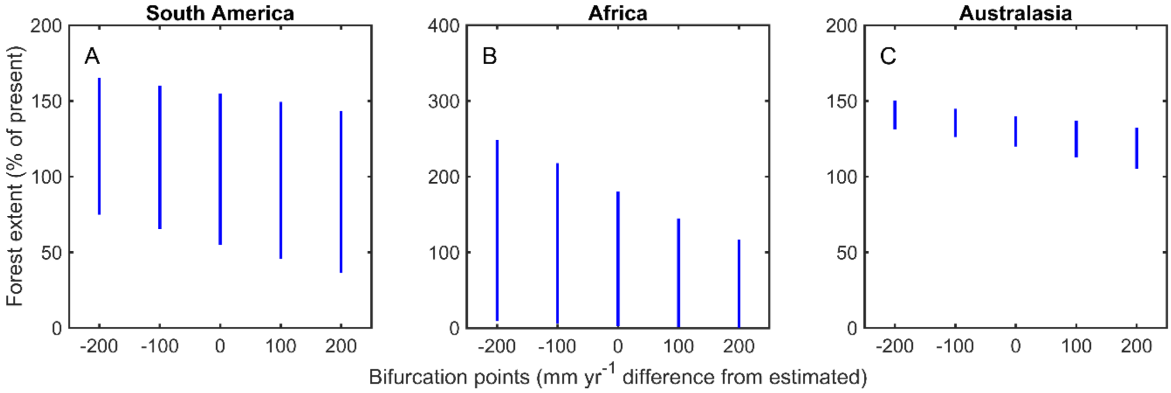

Supplementary Figure 19: Sensitivity of forest hysteresis (% of present extent) to bifurcation points in forest cover (mm yr^{-1} difference from estimated). The range of the blue bars depicts the hysteresis for -200, -100, 0, 100, and 200 mm yr^{-1} higher-than-estimated bifurcation points (Supplementary Figs. 1−3). Results are based on simulations for 2003 only. A) South America. B) Africa. C) Australasia.

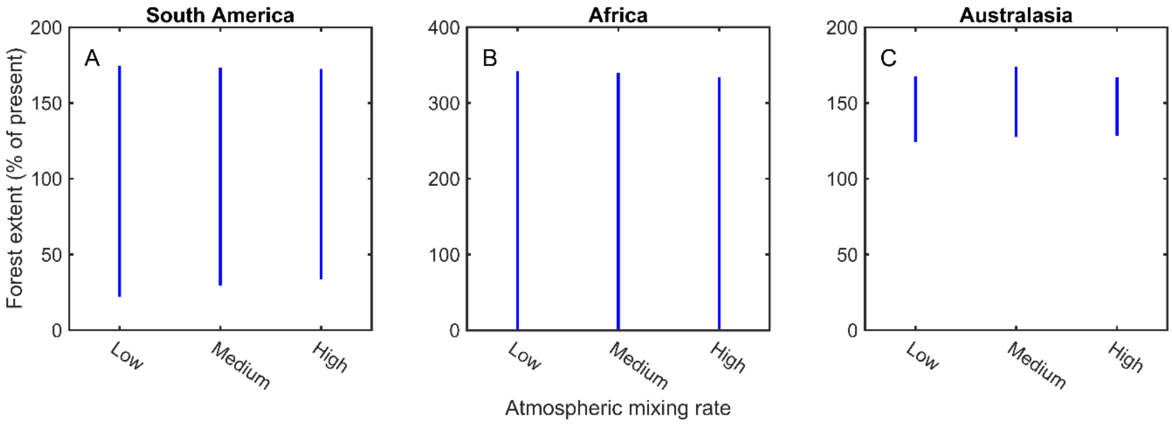

Supplementary Figure 20: Sensitivity of forest hysteresis (% of present extent) to different vertical mixing assumptions during atmospheric moisture tracking. The range of the blue bars depicts the hysteresis for low (mixing every 120 hours), medium (mixing every 24 hours), and high atmospheric mixing (mixing every hour; also see Methods). Results are based on simulations for 2003 only and were done at 0.5º. A) South America. B) Africa. C) Australasia.

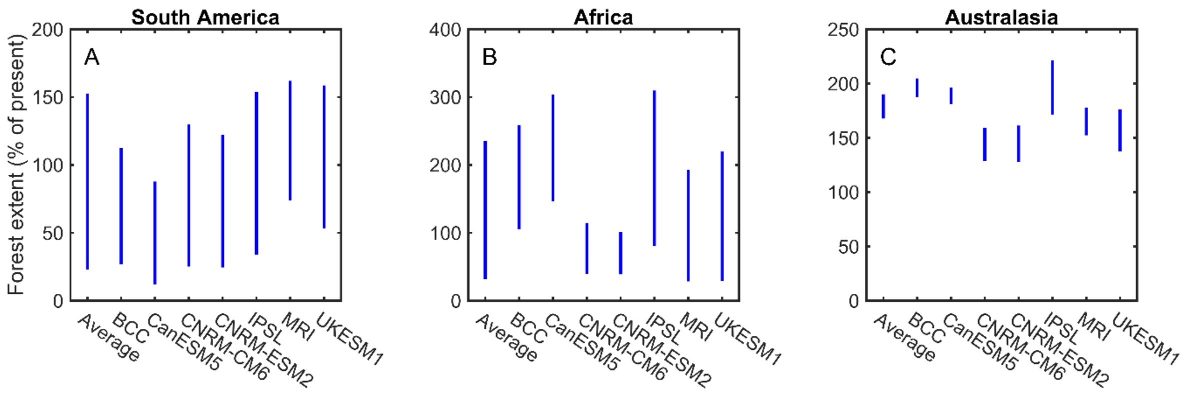

Supplementary Figure 21: Sensitivity of forest hysteresis (% of present extent) to different CMIP6 models. The range of the blue bars depicts the hysteresis for each of the models used and their average (see Methods). Results are based on simulations for 2003 only. A) South America. B) Africa. C) Australasia.

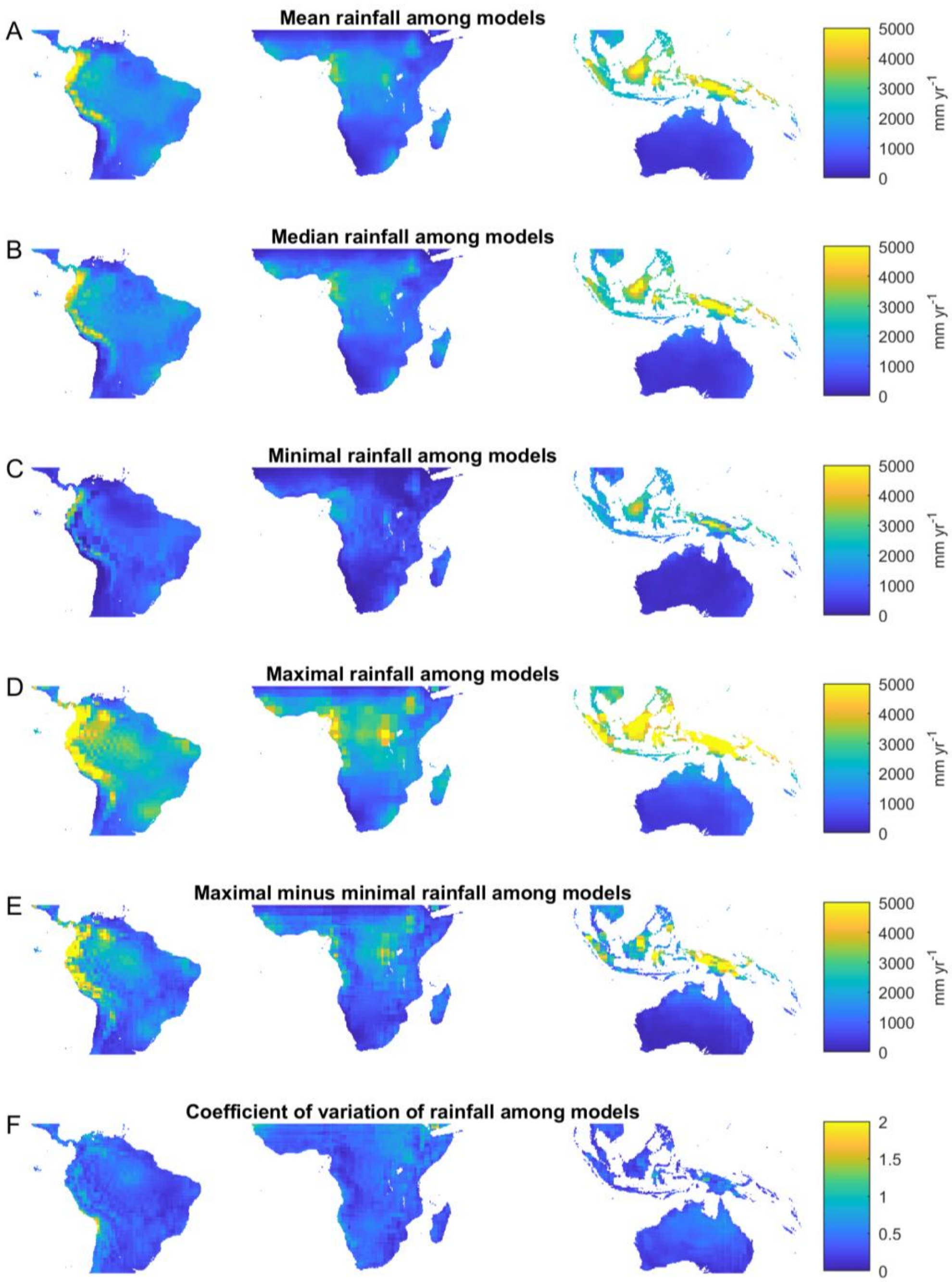

Supplementary Figure 22: Statistics of mean annual rainfall and their differences among the CMIP6 models. A) Mean rainfall among the models (mm yr^{-1}). B) Median rainfall among the models (mm yr^{-1}). C) Minimal rainfall among the models (mm yr^{-1}). D) Maximal rainfall among the models (mm yr^{-1}). E) Maximal minus minimal rainfall among the models (mm yr^{-1}). F) Coefficient of variation of rainfall among the models (-).

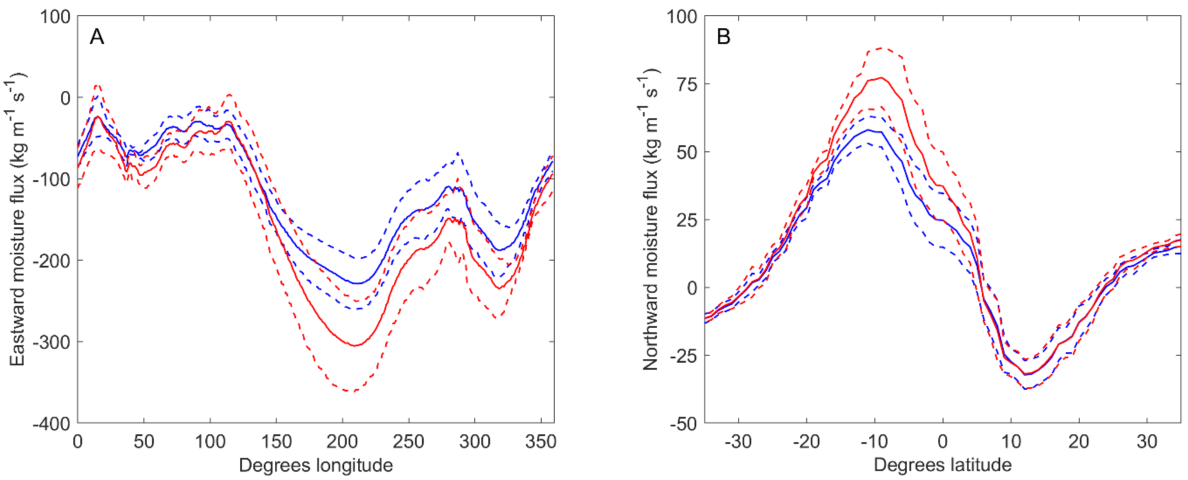

Supplementary Figure 23: The eastward and northward vertically integrated moisture fluxes (kg m^{-1} s^{-1}) in the CMIP6 models used for this study. Blue: 2015−2020, with the solid lines being the means and the dashed lines the means ± one standard deviation. Red: 2095−2100, with the solid lines being the mean sand the dashed lines the means ± one standard deviation. A) Net eastward moisture flux for the tropics across longitudes. B) Net northward moisture flux for the tropics across longitudes.
