# Supplementary material for: Hysteresis of tropical forests in the 21st century
Source: Nat Commun. 2020 Oct 5;11:4978. doi: 10.1038/s41467-020-18728-7 (PMC7536390; doi:10.1038/s41467-020-18728-7)
Supplement: Supplementary file 3 — Description of Additional Supplementary Files [file 41467_2020_18728_MOESM3_ESM.pdf]

Description of Additional Supplementary Files

File Name: Supplementary Data 1
Description: The data to reproduce Figure 2.
