# Supplementary material for: Hysteresis of tropical forests in the 21st century
Source: Nat Commun. 2020 Oct 5;11:4978. doi: 10.1038/s41467-020-18728-7 (PMC7536390; doi:10.1038/s41467-020-18728-7)
Supplement: Supplementary file 5 — Reporting Summary [file 41467_2020_18728_MOESM5_ESM.pdf]

nature research

Corresponding author(s): Arie Staal

Last updated by author(s): Aug 18, 2020

Reporting Summary

Nature Research wishes to improve the reproducibility of the work that we publish. This form provides structure for consistency and transparency in reporting. For further information on Nature Research policies, see Authors & Referees and the Editorial Policy Checklist.

Statistics

For all statistical analyses, confirm that the following items are present in the figure legend, table legend, main text, or Methods section.

n/a	Confirmed	
☒	☐	The exact sample size (*n*) for each experimental group/condition, given as a discrete number and unit of measurement
☒	☐	A statement on whether measurements were taken from distinct samples or whether the same sample was measured repeatedly
☒	☐	The statistical test(s) used AND whether they are one- or two-sided<br>*Only common tests should be described solely by name; describe more complex techniques in the Methods section.*
☒	☐	A description of all covariates tested
☒	☐	A description of any assumptions or corrections, such as tests of normality and adjustment for multiple comparisons
☐	☒	A full description of the statistical parameters including central tendency (e.g. means) or other basic estimates (e.g. regression coefficient) AND variation (e.g. standard deviation) or associated estimates of uncertainty (e.g. confidence intervals)
☒	☐	For null hypothesis testing, the test statistic (e.g. *F*, *t*, *r*) with confidence intervals, effect sizes, degrees of freedom and *P* value noted<br>*Give P values as exact values whenever suitable.*
☒	☐	For Bayesian analysis, information on the choice of priors and Markov chain Monte Carlo settings
☒	☐	For hierarchical and complex designs, identification of the appropriate level for tests and full reporting of outcomes
☒	☐	Estimates of effect sizes (e.g. Cohen's *d*, Pearson's *r*), indicating how they were calculated

Our web collection on statistics for biologists contains articles on many of the points above.

Software and code

Policy information about availability of computer code

Data collection	All data used are publicly available. No software was to used to collect data.
Data analysis	All data analyses were carried out in MATLAB R2019a. Figure 2 was made using Matplotlib 2.2.5.

For manuscripts utilizing custom algorithms or software that are central to the research but not yet described in published literature, software must be made available to editors/reviewers. We strongly encourage code deposition in a community repository (e.g. GitHub). See the Nature Research guidelines for submitting code & software for further information.

Data

Policy information about availability of data

All manuscripts must include a data availability statement. This statement should provide the following information, where applicable:

- Accession codes, unique identifiers, or web links for publicly available datasets
- A list of figures that have associated raw data
- A description of any restrictions on data availability

The Landsat tree cover data are available at https://e4ftl01.cr.usgs.gov/MEASURES/GFCC30TC.003/. The PCR-GLOBWB hydrological model experiment was forced with WATCH ERA-Interim data available for download at ftp://ftp.iiasa.ac.at/. Further forcing data of the model are available for download at https://zenodo.org/record/1045339#.XzZlejVcJhF. The moisture tracking model used ERA5 data available for download at https://www.ecmwf.int/en/forecasts/datasets/reanalysis-datasets/era5 and GLDAS2 data available for download at https://disc.sci.gsfc.nasa.gov/datasets?keywords=GLDAS. FLUXCOM data can be downloaded from http://fluxcom.org/EF-Download/. ESA GlobCover data can be downloaded at http://due.esrin.esa.int/page_globcover.php. CMIP6 model output as downloaded from https://esgf-node.llnl.gov/projects/cmip6/. The data for Figure 2 are available as online supplementary information (Dataset 1). For further requests, please contact the corresponding author.

Field-specific reporting

Please select the one below that is the best fit for your research. If you are not sure, read the appropriate sections before making your selection.

☐ Life sciences ☐ Behavioural & social sciences ☒ Ecological, evolutionary & environmental sciences

For a reference copy of the document with all sections, see nature.com/documents/nr-reporting-summary-flat.pdf

Ecological, evolutionary & environmental sciences study design

All studies must disclose on these points even when the disclosure is negative.

Study description	It is a modelling study requiring climatic and land-cover data for the tropics, which are publicly available (see links above). In addition, data analysis on tree cover data (a remote sensing product) was performed (see below).
Research sample	For the potential analysis using tree cover data (Landsat tree cover data for 2000 on 30 m resolution) to estimate bifurcation points against climatic variables, sampling was done. For each of the three continents studied (South America, Africa, and Australasia), we randomly sampled one million points. These data are available here: https://e4ftl01.cr.usgs.gov/MEASURES/GFCC30TC.003/
Sampling strategy	The tree cover data have global coverage at a resolution of 30 m. We decided to sample one million data points per continent to keep the analysis consistent among continents. One million data points is more than sufficient to obtain bifurcation plots so bifurcation points could be identified, while sampling reduces possible effects of spatial autocorrelation. It is also in the same order of magnitude as previous studies doing similar analyses on remotely sensed tree cover data.
Data collection	The data were collated by the corresponding author.
Timing and spatial scale	The data represent tree cover in the year 2000. The study area was the tropics (South America, Africa and Australasia between 15 degrees North and 35 degrees South. The spatial resolution of the data is 30 m. Tree cover distributions were related to the climatic variables mean annual rainfall and MCWD, which were obtained from GLDAS 2.0 data for 1970-1999 at 0.25 degree resolution.
Data exclusions	We masked out the tree cover in human-used areas, water bodies and bare ground using the ESA GlobCover dataset (values 11-30 and 190 or above). The GlobCover data at 300 m resolution are available here: http://due.esrin.esa.int/page_globcover.php. The exclusion was pre-established.
Reproducibility	No experiments were carried out. To reproduce the analyses, see the Methods and the Data availability and Code availability statements.
Randomization	The analysis were separated among the continents, as tree cover may have different responses to climatic variables.
Blinding	Sampling was done randomly, but apart from that no blinding occurred.

Did the study involve field work? ☐ Yes ☒ No

Reporting for specific materials, systems and methods

We require information from authors about some types of materials, experimental systems and methods used in many studies. Here, indicate whether each material, system or method listed is relevant to your study. If you are not sure if a list item applies to your research, read the appropriate section before selecting a response.

Materials & experimental systems

n/a	Involved in the study
☒	☐ Antibodies
☒	☐ Eukaryotic cell lines
☒	☐ Palaeontology
☒	☐ Animals and other organisms
☒	☐ Human research participants
☒	☐ Clinical data

Methods

n/a	Involved in the study
☒	☐ ChIP-seq
☒	☐ Flow cytometry
☒	☐ MRI-based neuroimaging
